# Silver nanoparticle conductive inks: synthesis, characterization, and fabrication of inkjet-printed flexible electrodes

**DOI:** 10.1038/s41598-020-65698-3

**Published:** 2020-06-01

**Authors:** Iara J. Fernandes, Angélica F. Aroche, Ariadna Schuck, Paola Lamberty, Celso R. Peter, Willyan Hasenkamp, Tatiana L. A. C. Rocha

**Affiliations:** itt Chip - Institute of Technology on Semiconductors, Universidade do Vale do Rio dos Sinos, São Leopoldo, 93022-750 Brazil

**Keywords:** Biomedical engineering, Chemical engineering, Electrical and electronic engineering

## Abstract

Flexible electronics can be developed with a low-cost and simple fabrication process while being environmentally friendly. Conductive silver inks have been the most applied material in flexible substrates. This study evaluated the performance of different conductive ink formulations using silver nanoparticles by studying the material properties, the inkjet printing process, and application based on electrical impedance spectroscopy using a buffer solution. Silver nanoparticles synthesis was carried out through chemical reduction of silver nitrate; then, seven conductive ink formulations were produced. Properties such as resistivity, viscosity, surface tension, adhesion, inkjet printability of the inks, and electrical impedance of the printed electrodes were investigated. Curing temperature directly influenced the electrical properties of the inks. The resistivity obtained varied from 3.3 × 10^0^ to 5.6 × 10^−06^ Ω.cm. Viscosity ranged from 3.7 to 7.4 mPa.s, which is suitable for inkjet printing fabrication. By using a buffer solution as an analyte, the printed electrode pairs presented electrical impedance lower than 200 Ω for all the proposed designs, demonstrating the potential of the formulated inks for utilization in flexible electronic devices for biological sensing applications.

## Introduction

Inkjet printing has been investigated as an alternative production tool for the fabrication of conductive elements and devices in the field of flexible electronics. This fabrication technique deposits particles of the material with desirable electrical properties onto a substrate, after which, the printed pattern is converted into conductive elements^1^. There are benefits related to the inkjet printing, namely, a simple fabrication process, low cost, reduction of material waste, and excellent adequacy to several substrates^2–4^. This printing process involves the storage of ink in a cartridge and the ejection of an exact amount of material through the nozzles^5^. Therefore, the fabrication of flexible circuits, sensors, and other printed materials represents a great technological advancement compared with other standard methods, such as drop casting or stamping^6^.

Silver remains one of the best options for application as a conductive ink and adhesive, compared to other electrically conductive fillers. This is mainly due to its high electrical and thermal conductivity, chemical stability, relatively low cost (compared to gold or graphene, for example), and the ability of its oxide form to conduct electricity^2^. Additionally, silver nanoparticles have a low melting point, which promotes the generation of conductive thin films in relatively low temperatures, this is vital to applications in flexible substrates, such polymers and papers^4,7,8^. Different methods can be used for the synthesis and stabilization of silver nanoparticles. One of the most popular approaches is chemical reduction, using a variety of organic and inorganic reducing agents^7,9^. Depending on the method used silver nanoparticles can be fabricated with different morphologies, sizes, shapes, and concentrations^10^.

Conductive inks are generally formulated with metallic particles (e.g. silver, copper, and gold) or carbon particles (such as graphene and carbon nanotubes) in a retention matrix. The matrix in this application needs to be insulate or a weak conductor. In order to create a contact path with the conductive particles, it is necessary that the volume of the matrix be reduced either with a curing or evaporation process, also called the sintering process^2^. Organic dispersant and stabilizers are added into the silver nanoparticle conductive ink formulation to prevent agglomeration caused by the high surface energy of these nanoparticles. Sintering decomposes these organic agents, which are used to encapsulate the nanoparticles, allowing these particles to interact. Although there are many sintering techniques, such as thermal, chemical, electric, and laser sintering, these methods result in extra costs and time after the printing process^11^. As an alternative, substrates surface which have been coated with cationic polymers have been used. These cationic polymers spontaneously sinter the particles. This sudden reaction occurs due to the presence of chloride ions in the coating, which decapsulates the nanoparticle from the deagglomerating agents, promoting the sintering of the particles. Kodak and Epson photo papers both contain this type of coated surface^11–13^.

Nanoparticle conductive ink has its particles suspended in water or organic solvents, such as ethylene glycol, toluene, or cyclohexane. The solvent used evaporates after the ink deposition through the inkjet process, but not quickly enough to dry in the ejection nozzle, hindering the ejection of the fluid^14^. Ethanolamine is added in a small amount in the ink formulation to prevent the nozzle from clogging^15^. Significant properties of the ink fluid are viscosity and surface tension. Depending on the printing machine, these properties vary, but in general, for inkjet printing, viscosity ranges from 1 to 25 mPa.s and surface tension from 25 to 50 mN/m^14^.

There are many works that use conductive inks to print electrodes on flexible substrates for biosensor applications^16–19^. Besides the simple large-area fabrication technique, i.e. conventional printing technologies, those electrodes can be easily patterned to integrate microfluidic channels enabling the investigation of the electrical properties in biological samples^17^. The reactions that occur in the channel with the presence of a bioreceptor, e.g. reagent or enzyme, change the properties of the analyte, such as electrical conductivity and impedance^20–22^.

With that in mind, this study aimed to develop different conductive ink formulations using silver nanoparticles, investigate the formulation properties, fabrication process performance, and the application as electrodes to detect biochemical reactions based on electrical impedance using a buffer solution.

## Methods

### Materials

Silver nitrate (Sigma Aldrich, 99%), polyvinylpyrrolidone (Sigma Aldrich, MW = 111.1), sodium borohydride (Sigma Aldrich, 97%), ethylene glycol (Vetec, 99%), ethanol (Química Moderna, 95%), ethanolamine (Dinâmica Química, 99%), Solsperse 20000 (Lubrizol, PA), sodium alginate (Sigma Aldrich, low viscosity), hydroxyethyl-cellulose (Sigma Aldrich, MW = 90) were all used as received. The PEDOT:PSS (1:2.5) dispersion was made by mixing 3,4-ethylenedioxythiophene (EDOT, Sigma Aldrich 97%, MW = 142.18), ammonium persulfate (APS, Synth 98%, MW = 220.19) and polystyrene sulfonate (PSS, Sigma Aldrich MW = 75k), using iron sulfate III (Sigma Aldrich 97%) as the reaction initiator. The molar ratio of the reaction was 2:1:0.0125 (EDOT:APS:PSS). The preparation method follow the methodology described by Rodrigues *et al*.^23^.

### Silver nanoparticle synthesis

Silver nanoparticles were obtained through chemical reduction of silver nitrate (AgNO_3_) by sodium borohydride (NaBH_4_) in the presence of polyvinylpyrrolidone (PVP, MW = 111.1)^24^. In a typical synthesis, at room temperature, 8 mL of NaBH_4_ (0.529 M) solution was added at once to a reaction mixture containing 0.006 mols of AgNO_3_ and 0.009 mols of PVP in 100 mL of deionized water; resulting in an immediate change of solution color from dark yellow/brown. The synthesis was carried out inside a glass beaker of 600 mL. Subsequently, the resulting solution was stirred for 8 minutes (99% rpm, magnetic stirrer, TECNAL TE-0851) and filtered (Quantitative filter paper 8 μm); lastly, silver nanoparticles were separated by centrifugation (15,000 rpm for 1 hour) in a Hitachi centrifuge (CF15RN) using 50 mL PP tubes.

### Conductive inks formulation

Different conductive ink formulations were created from the standard synthesis method for silver nanoparticles. Table 1 shows the solvents used in each ink formulation, volumetric ratio, and the weight percentage of silver nanoparticles present. Inks were formulated with a total volume of 6 mL. Ethylene glycol was used in the formulations to prevent the inks from drying too quickly on the nozzles^25^. Ethanolamine was used as a surfactant and emulsifier, bringing durability and stability to the inks. Dispersant containing polyesters (Solsperse 20000) was added in a small amount to improve ink stability. The other components were used as solvents or as agents to increase viscosity. As a reference, a silver nanoparticle dispersion in ethanol (named formulation I-1) was elaborated. I-2 was formulate using a sodium alginate solution, ethylene glycol, water, ethanol and hyperdispersant. Solutions of hydroxyethyl-cellulose for I-3 and I-5 formulation were prepared with water and ethanol in a volumetric ratio of 1:1. For comparison, I-4 was elaborated using only ethylene glycol, water, ethanol and hyperdispersant. I-6 and I-7 formulations were elaborated with silver nanoparticles from two standard syntheses.

**Table 1:** Silver nanoparticles based conductive ink formulations.

Ink Label	Solvents	Volume Ratio of Solvents	AgNP (wt %)
I-1	Ethanol	1	~ 8
I-2	Sodium alginate solution 1%, ethylene glycol, water, ethanol, hyperdispersant (Solsperse 20000)	1:1:3.7:4.5:0.01	~ 8
I-3	Hydroxyethyl-cellulose solution 0.3%, water, hyperdispersant (Solsperse 20000)	5:1:0.006	~ 8
I-4	Ethylene glycol, water, ethanol, hyperdispersant (Solsperse 20000)	1:2:2:0.005	~ 8
I-5	Hydroxyethyl-cellulose solution 0.5%, water, ethtylene glycol, ethanolamine, hyperdispersant (Solsperse 20000)	3.8:1:1.2:0.04:0.006	~ 8
I-6	Ethylene glycol, water, ethanol, ethanolamine, hyperdispersant (Solsperse 20000)	1:2:2:0.03:0.005	~ 16
I-7	Ethylene glycol, ethanol, PEDOT:PSS dispersion, water, ethanolamine, hyperdispersant (Solsperse 20000)	1.2:2.4:1.4:1:0.04:0.006	~ 16

### Characterization of silver nanoparticles and ink formulations

Nanoparticle sizes were estimated by laser size distribution analyzer (Dynamic light scattering (DLS) – Nanophox Sympatec). This analysis was performed on ink I-1, which was formulated with the nanoparticles dispersed in ethanol. Ink viscosity was analyzed in a viscometer Brookfield (RV DV II, spindle 18) at room temperature and 200 rpm. Surface tension was measured using an optic tensiometer THETA (model OneAttension), in this analysis, a pendant drop from the fluid, measuring 4 μL, was created on the tip of the needle. The temperature for surface tension measurement was between 23–24 °C. Electrical resistivity was measured by four-point probe method (Pro4-4000 Signatone), this electrical property was preliminary studied in all formulated inks on a glass substrate (microscope slides), and then, for the inks I-6 and I-7, on a photo paper (EPSON – Glossy photo paper). For the resistivity measurement on the glass substrate drop casting technique was used, for that, with a micropipette, 60 μL of ink was deposited on the substrate in order to obtain a metallic film; a vinyl tape was used to create a circular shape (Ø = 20 mm) of the ink on the substrate (Figure S1). After the ink deposition, the ink was cured in different temperatures (150, 200, and 300 °C for 30 minutes), and for each temperature a new sample of the same ink was used. The microscope slides were previously cleaned using plasma (Oxygen gas, flow of 100 sccm, 200 W, for 10 min - Plasma Etch model PE-200). For the resistivity measurement on the photo paper only ink I-6 and ink I-7 were used in the characterization, and a square printed pattern was utilized (20 mm of side). These two inks were each printed on the photo paper using a Dimatix printer, and the parameters used are described in the following section (*Inkjet Printing Process*). The D-500 KLA-Tencor profilometer was used to determine the thickness of the ink samples deposited on the glass substrate. Scanning electron microscope ZEISS EVO LS15 was used to measure the thickness of the inkjet-printed designs onto the photo paper, backscattering detectors and 15 kV were applied. The structural investigation of the conductive ink was carried out by field emission scanning electron microscopy with secondary electron detectors at 15 kV (FEI XL830 Dualbeam Workstation). The adhesion test was determined using ASTM D3359 (ASTM 2009) on the photo paper, glass (microscope slides), and PET (Melinex) substrates. For the adhesion test, the inks were deposited on the substrates following the same procedures as for the resistivity measurements with the exception of the sample shape, which was square (Figure S1). Both glass and PET substrate were cleaned before the test using the plasma procedure delineated previously. In addition, to adhesion test sinterization at 50 °C for 30 min was performed on the inks deposited on photo paper, while on glass and PET substrates, the sinterization was carried out at 150 °C for 30 min.

### Inkjet printing process

To verify the inkjet printing process and evaluate each formulation performance, the inks were used for printing from a FUJIFILM Dimatix DMP 2850 in piezoelectric cartridges of 10 pL, with exception of ink I-1. Before the printing tests, the inks were previously filtered with a 0.20 µm syringe filter to avoid obstruction of nozzles with the aggregation of particles. After filtration, the fluids were placed inside a vacuum desiccator for 30 min to remove bubbles. Glossy photo paper (EPSON) was used as the substrate. Printing parameters for printability tests varied, between 7 to 16 nozzles were used, firing voltage of 17 to 23 V, maximum jetting frequency of 2 to 10 kHz, and cartridge print height of 1 mm. The parameters used for the samples printed for resistivity measurements of ink I-6 and I-7 as well as for the electrode patterns printed with these two inks are as follow: 7 nozzles, firing voltage of 20 V, maximum jetting frequency of 2 kHz, and cartridge print height of 1 mm. One printing layer and a drop spacing of 10 μm (2540 dpi) were used for printability test of the inks, samples printed for the resistivity characterization, and printing of electrodes onto photopaper. All the measurements carried out on the inks printed were performed at least after 24 h of the deposition, and the printed patterns were totally dried after this period; there was no sintering or drying process done after the printing of the inks.

### Electrochemical characterization of the inkjet-printed electrodes

**Figure 1:**
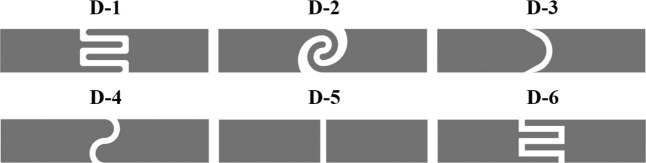
Electrode shapes designed for the electrical characterization experiment (Width: 27.2 mm; Height: 5.6 mm; Gap: 0.8 mm).

Electrodes were designed using AutoCAD software and printed following the method mentioned in previous subsection. For this experiment, the ink formulations that presented exceptional electrical and mechanical properties were selected. For the inkjet-printed electrodes characterization, electrical impedance spectroscopy (EIS) measurements were carried out to verify each electrode pair performance using 1× phosphate-buffered saline (PBS; pH 7.4) solution as an electrolyte. Measurements were performed in a two-electrode setup using a commercially available Metrohm Autolab PGSTAT302 potentiostat system (Figure S2). For reference values, 10 µL of buffer solution was inserted in the plotted vinyl well (Ø = 5 mm), and the impedance response was recorded with an input AC voltage at 10 mV in a frequency range of 0.1 Hz to 100 kHz. Different electrode designs (Fig. 1) were considered with a modification in the shape of the contact region with the analyte. The electrical characterization was repeated for each shape proposed.

## Results and Discussion

**Figure 2:**
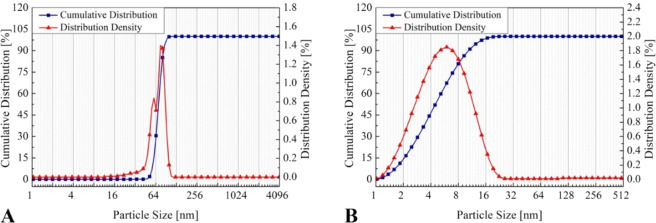
The particle size distribution of silver nanoparticles obtained (**A**) and supernatant (**B**).

Figure 2 shows the particle size distribution of silver nanoparticles synthesized (Fig. 2A), and of discarded supernatant (Fig. 2B). The analysis was performed only on ink I-1. The mean particle size of silver nanoparticles synthesized was 70 ± 1 nm with 90% of nanoparticles having a diameter less than 84 nm. Supernatant showed a mean particle size of 5 ± 0.1 nm with 90% of nanoparticle with diameter under 10 nm. It indicates that the centrifugation process, even with a high rotation (15,000 rpm), is not capable of separating nanoparticles with a size less than 10 nm, segregating only the bigger nanoparticles (≥50 nm). The size and homogeneity of nanoparticles interferes directly in the quality of the formed conductive layers^26^. It is desirable to obtain small nanoparticles to avoid the nozzle clogging during the inkjet printing process. However, the smaller the nanoparticle formed, the more complex the separation process through centrifugation without irreversible agglomeration.

Kosmala *et al*.^27^ explain that during the synthesis process, the primary nanoparticles formed strongly attract each other through physical forces that are present due to high surface area, creating bigger particles immediately^27^. Polavarapu *et al*.^24^, the reference used for the silver nanoparticles synthesis, produced nanoparticles with sizes varying between 15 to 30 nm, smaller than those obtained in this paper. This difference may be a result of the rotation of the centrifugation, which was higher than the rotation used in the reference (5,000 rpm), promoting agglomeration^24^. The rotation speed applied was experimentally defined, and it was observed that lower rotation results in more time required for nanoparticles to sediment. Using 15,000 rpm, it was possible to achieve high yield of nanoparticle content with only 1 h of centrifugation.

All properties of the material are affected by the ink quality, such as evaporation and film homogeneity. This means that each ink formulation may be suitable for different applications. In general, to formulate conductive inks, the most commonly used materials are particles of silver dispersed in a proper mean, which allow good ink ejection^28^. Considering that organic materials were applied as solvents, and humectant agents used to increase viscosity are non-conducting, the sample I-1 was formulated only with ethanol to obtain a minimum attainable resistivity. However, this sample was not used in the printing tests because it did not have the required characteristics for printing, as the lack of humectant could clog the nozzles.

According to Shen *et al*.^13^, inkjet printing technique involves the production of small droplets of liquids and their deposition on a specific location of the substrate^13^. In this process, the surface tension and viscosity are the physical properties of the ink that define the jet and the droplet behavior. Table 2 shows the results of viscosity obtained using a viscosimeter Brookfield at room temperature and 200 rpm.

**Table 2:** Viscosity of the different ink formulations.

Ink Formulation	Viscosity (mPa.s)
I-1	2.85
I-2	3.69
I-3	5.33
I-4	4.17
I-5	7.41
I-6	5.08
I-7	5.25

Dimatix Fujifilm DMP 2850 user guide suggests using a fluid viscosity in a range between 10 to 12 mPa.s^29^. According to a review carried out by Rao *et al*.^7^, inkjet printing requires relatively low ink viscosity, in the range of 2.51 to 4.03 mPa.s, besides a complex mixture of solvents with suitable concentration to avoid clogging the nozzles^7^. The lowest value of viscosity was found on the I-1 formulation using solely ethanol as reference. The other inks formulated in this work exhibited viscosities varying from 3.69 to 7.41 mPa.s, similar values to the results reported by Rao *et al*.^7^. These viscosities could be adequate for inkjet printing applications. The highest viscosity obtained was observed on ink I-5 (7.41 mPa.s) due to the concentration of the hydroxyethyl-cellulose solution. In addition, printing tests confirmed that the obtained viscosities offer great printing quality.

Surface tension is caused by intermolecular forces that exist in liquids, and similar to viscosity, it is an important parameter for inkjet printing applications^13^. If the fluid presents a very low surface tension, it can leak from the nozzles, whereas if the surface tension is very high, the fluid can be expelled from the cartridge. Table 3 shows the results for the surface tension which varies between 32.5 and 40.8 mN/m for the different formulations. According to Cummins and Desmulliez^14^, inkjet printing technique typically requires a surface tension between 25 to 50 mN/m. The Dimatix Fujifilm user guide recommends a surface tension of 33 ± 3.5 mN/m^29^. Different authors developed inks with a surface tension inside a range of 33 to 36 mN/m^30–32^. Thus, in comparison with these authors and the printer user guide, it is inferred that the inks which are more suitable to inkjet printing would be formulations I-2, I-6, and I-7.

**Table 3:** Surface tension for the different ink formulations.

Ink Formulation	Surface tension (mN/m)
I-1	32.5
I-2	36.8
I-3	40.8
I-4	39.8
I-5	39.7
I-6	35.4
I-7	35.3

**Figure 3:**
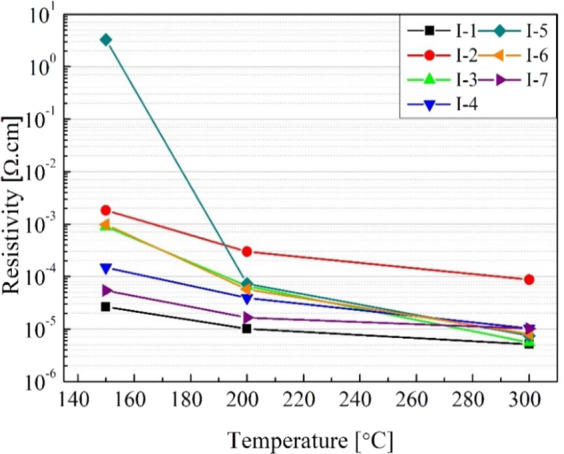
Resistivity of the different formulations cured at temperatures 150, 200, and 300 °C.

**Figure 4:**
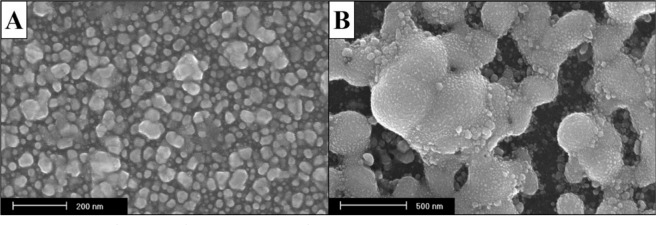
FE-SEM surface analysis of ink samples at room temperature (**A**) and after the sintering process at 300 °C (**B**).

Resistivity data is displayed in Fig. 3, where vertical axis values are expressed on a logarithmic scale in order to generate a better view of the results. The resistivities of the inks at all curing temperatures varied from 3.3 × 10^0^ to 5.6 × 10^−06^ Ω·cm. The lowest resistivity obtained at a temperature of 150 °C was observed on formulation I-1, which contained only silver nanoparticles and ethanol. Variation of temperature in the sintering process is one of the most important parameters that affect the silver nanoparticles inks^9^. A clear decrease in resistivity is noticeable as curing temperature increases. The reduction of the resistivity occurs due to two main factors. Firstly, the sintering process of silver nanoparticles causes a necking formation between the nanoparticles as shown in Fig. 4, and second, the decomposition of the organic solvents present in the formulations. Figure 3 illustrates that even at the highest temperature (300 °C, 30 min), the sample I-2 has the highest resistivity. This can be explained by the fact that the melting point of sodium alginate is greater than 300 °C. The lowest resistivity observed was from formulation I-3 at 300 °C, 30 min (5.6 × 10^−06^ Ω·cm). Regardless ink I-1 which was developed only as a reference, for the lowest curing temperature (150 °C, 30 min), the best resistivity was obtained using the ink I-7 (5.4 × 10^−05^ Ω·cm). Mo *et al*.^33^ wrote in their review article that lowest resistivities were achieved in the range of 2.2 × 10^−06^ to 15.1 × 10^−06^ Ω·cm for methods using different sintering temperatures, sintering process, concentrations, shapes, and sizes of nanoparticles. Thus, it is inferred that the resistivities achieved in this work are satisfactory for different applications in comparison with the studies presented in literature.

As for the layers deposited, there were neither cracks on the inks nor on the glass substrate after sintering the inks at all temperatures. It was observed that at 300 °C all inks had their color changed to a lighter shade. Regarding their surface appearance through all temperatures, ink I-1 had the smoothest surface, which is likely due to the fact that the ink was comprised of only ethanol that rapidly evaporated. Conversely, inks I-3 and I-5 had the surface with the roughest aspects, such as wrinkles. These two inks have Hydroxyethyl-cellulose (HEC) solution in great quantity in their formulation, and HEC solution may have played a large role in their appearance since this reagent is a gel thickening agent. The presence of this agent promoted a rubber movement of the ink while being evaporated, which caused the wrinkles. The surface of the other inks, I-2, I-4, I-6, and I-7 were uneven, presenting some local concentration. While I-2 and I-4 surfaces spread towards the edges, inks I-6 and I-7 had their composition concentrated in the center region. Ink I-7 showed a slightly less concentration in the center region than ink I-6. The presence of ethanolamine in ink I-6 and PEDOT:PSS solution in ink I-7 may have been the factor which differentiated their inks evaporating behavior compared to inks I-2 and I-4, which are also comprised with ethylene glycol.

The adhesion results are summarized on Table 4, and it is noticeable that sample I-1 showed the best adhesion for all substrates. After solvent evaporation, only a metallic layer remained, improving the adhesion of ink I-1. Additionally, sample I-5 presented a better adhesion on glass, remaining intact after the test. On photo paper substrate, all ink samples showed ideal adhesion behavior, which may be a consequence of its surface treatment. The adhesion characteristic of the inks is an important parameter to select the most suitable formulation or substrate for all possible applications. In order to use an ink on a substrate, the ink has to present adhesion onto the substrate. Photo paper and PET substrates are largely used as substrates for flexible electronics^9,11,13,15,34–39^; since PET showed lower ink adhesion values than photo paper for the inks developed in this work, printing tests were performed only on photo paper.

**Table 4:** Adhesion test in different substrates.

Ink Formulation	Glass substrate adhesion	PET substrate adhesion	Epson photo paper adhesion
I-1	4	5	5
I-2	2	2	5
I-3	1	1	5
I-4	4	3	5
I-5	5	3	5
I-6	3	1	5
I-7	3	1	5

Classification 5 represents excellent adhesion with 0% of removed area, and classification 0 represents very poor adhesion with removed area greater than 65%.

Regarding printability, inks I-2, I-4, I-5, I-6, and I-7 were able to be expelled from the cartridge, but ink I-3 dried in the nozzles and consequently clogged the cartridge. The absence of ethylene glycol in ink I-3 formulation may be the reason for the ink drying quickly in the nozzles, as the other formulations which all had ethylene glycol did not show this behavior. Among the inks which did not dry in the nozzles, ink I-2 began to not be expelled during the printing process. The use of ethanolamine in formulation I-2 could prevent nozzles from being obstructed. Although ink I-4 was expelled from the cartridge, it presented a poor quality of the printed pattern, for instance, the edges were wavy, and a region with a separation design was connected. Ink I-4 is very similar to the ink I-6 formulation, but it has no ethanolamine, which may have influenced its printability performance.

**Figure 5:**
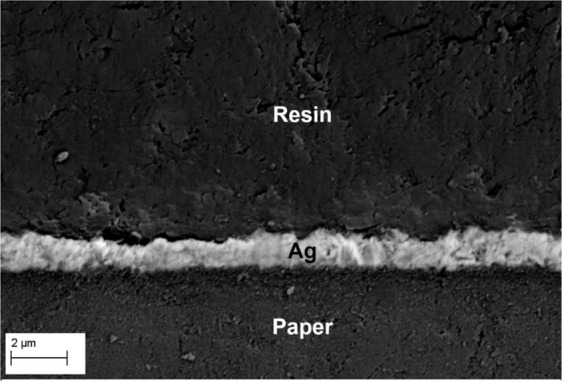
SEM cross-section of ink sample I-7 printed on a photo paper and embedded in an epoxy resin.

Inks I-5, I-6, and I-7 were the ones that demonstrated a conductive behavior on the photo paper with only one printed layer, but only inks I-6 and I-7 reproduced printing patterns with better quality, in other words, the patterns printed with ink I-6 and I-7 showed excellent details such as straight edges and separation of disconnected regions, on the other hand, the patterns printed with ink I-5 displayed poor details such as rounding of square edges, wavy edges, or even connection of separate regions. Considering the print quality and the resistivity values at the lowest curing temperature (150 °C, 30 min), thickness and resistivity tests were carried out on patterns printed on photo paper substrate using formulation I-6 and I-7. Figure 5 presents the SEM image of the ink I-7 cross-section. It is observed that the printed pattern has uniform thickness throughout the entire section, with a mean thickness of 1.3 µm.

Resistivity values were calculated based on the thickness measurements of the printed patterns on photo paper. Patterns printed using ink I-6 and I-7 showed resistivities of 1.6 × 10^−04^ and 5.7 × 10^−05^ Ω.cm, respectively. These resistivity values on photo paper are better than those obtained on a glass substrate with a curing temperature of 150 °C (Fig. 3). Some studies show that glossy photo paper (Epson) contains a surface coated with chloride ions which, during the silver dispersion deposition and absorption of the fluid vehicle, migrate to the silver film promoting decapsulation of silver nanoparticles from the deagglomeration agents, then assisting with the sintering process^11,12,33^.

The use of silver nanoparticle conductive ink on photo paper without thermal treatment were investigated by other authors. Magdassi *et al*.^12^ carried out a study using conductive ink printed on substrates with chloride ions^12^. The ink formulated by the team and printed on Epson photo paper reached resistivity of 6.8 × 10^−06^ Ω.cm at room temperature. The resistivity value achieved in Magdassi *et al*.^12^ is better than the values found for inks I-6 and I-7, for the lower the resistivity of an ink the better its conductivity; however, the low resistivity value in Magdassi *et al*.^12^ seems to be a result of the higher silver nanoparticles content (30 wt%) used, which is almost twice as much (16 wt%) used in this work. Huang *et al*.^38^ were able to fabricate a silver film pattern on photo paper with a resistivity of 12.6 × 10^−06^ Ω.cm, but this value represents an electrical property of 10 printed layers of the ink employed^38^. Herein, the resistivity of the pattern printed with inks I-6 and I-7 was obtained with only one layer. Shen *et al*.^13^ developed a conductive ink for flexible electronic applications^13^. In their study, the resistivity of two-printed layers achieved 1.4 × 10^−04^ Ω.cm at room temperature, similar to I-6 with only one printed layer, but inferior to the resistivity obtained with one layer of ink I-7 ^9^. Cao *et al*.^9^, reported a sheet resistance of 4 × 10^−03^ Ω·cm for an ink formulation containing 15 wt% of silver nanoparticles, with 40-layer printed pattern using a Dimatix printer. With this ink, the team was able to power an LED bulb using a 9 V battery, demonstrating the potential for electronic circuit applications. Therefore, taking into consideration the findings of Cao *et al*.^9^, inks I-6 and I-7 exhibit similar potential.

### Electrical impedance characterization of the printed electrodes

**Figure 6:**
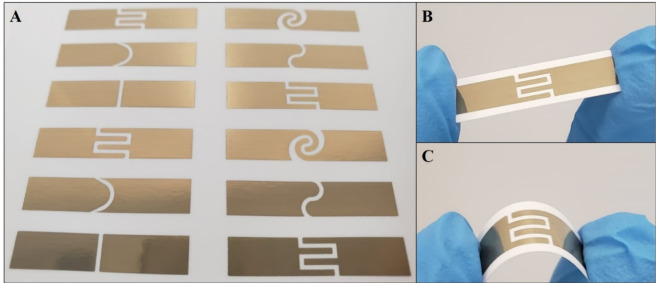
Different designs of the printed electrodes (**A**), with a closer view of design D-6 (**B**) and mechanical bending outwards (**C**) using the ink formulation I-7.

**Figure 7:**
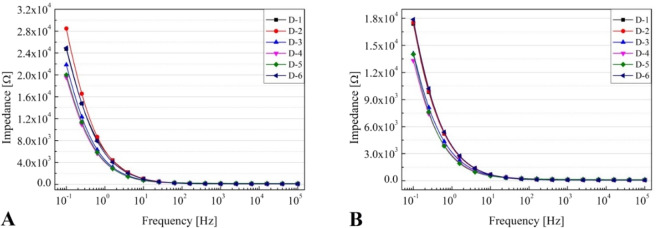
EIS response for a frequency range of 0.1 Hz to 10 kHz of the different printed electrodes pairs using the I-6 (**A**) and I-7 (**B**) ink formulations.

For the electrochemical characterization procedure, inks labeled as I-6 and I-7 were selected to fabricate the electrodes considering their outstanding performance in the conductivity experiment. In total, 18 electrode pairs (three of each proposed design) were printed for both ink formulations (I-6 and I-7) on a photo paper substrate, Fig. 6; the electrodes were tested after 24 h of being printed without going through any sintering procedure. Then, the EIS experiments were performed using the buffer solution, Fig. 7.

**Figure 8:**
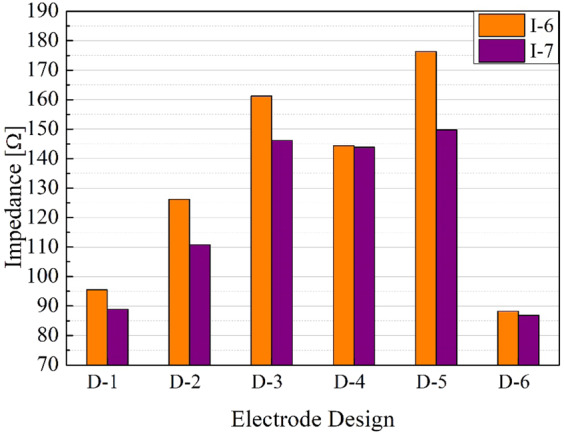
Electrical impedance measurement at 1 kHz for the different pairs of electrodes (D-1 to D-6) printed with ink formulations labeled I-6 and I-7.

The electrical impedance values were measured at 1 kHz for the different designs and compared in Fig. 8. The electrode pair design which showed the lowest impedance value was design D-6 for both ink formulations. However, when considering the conductive ink formulations, I-7 ink presented lower electrical impedance values (Table 5), which is consistent with the results obtained in the resistivity tests.

**Table 5:** Mean impedance values (n = 3) at 1 kHz for the different electrode designs using I-6 and I-7 ink formulations.

Electrode Design	Ink Formulation	Impedance [Ω]	SD [Ω]
D-1	I-6	95.50	5.39
	I-7	88.91	4.21
D-2	I-6	126.16	3.84
	I-7	110.78	1.89
D-3	I-6	161.22	28.79
	I-7	146.15	0.27
D-4	I-6	144.40	3.81
	I-7	143.87	9.56
D-5	I-6	176.38	5.53
	I-7	149.76	7.65
D-6	I-6	88.28	1.49
	I-7	86.80	1.59

**Figure 9:**
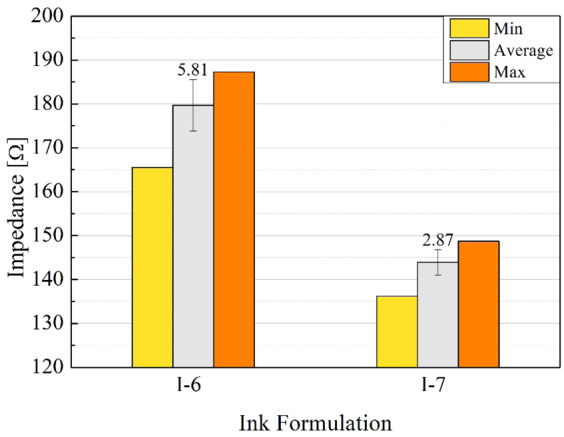
Mean impedance values (n = 30) at 1 kHz for the different ink formulations (I-6 and I-7) using the electrode design D-6.

In the next electrical characterization step, conductive electrodes with new dimensions (width: 27.4 mm; height: 5.8 mm; gap: 1 mm) were printed using design D-6 with inks I-7 and I-6 (n = 30 for each formulation). Following the trend of the other electrodes produced with this formulation, I-7 ink outperformed I-6, demonstrating good repeatability with a mean total impedance value of 143.91 Ω with a standard deviation (SD) of ± 2.87 Ω (Fig. 9).

## Conclusions

In this work, different formulations of silver nanoparticle conductive inks were developed to fabricate printed electrodes for biologic applications. The mean particle size of the silver nanoparticles was 70 ± 1 nm, with 90% of the nanoparticles having a diameter of less than 84 nm. The inks developed showed a viscosity varying between 3.69 and 7.41 mPa.s, which according to literature, would be adequate for inkjet printing. Ink I-1 presented good adhesion to glass, PET, and photo paper substrate, moreover, it was the ink that best adhered on PET substrate. Ink I-5 produced a good result for adhesion on a glass substrate. With regards to the photo paper substrate, all ink formulations offered excellent adhesion. The lowest resistivity at 300 °C was achieved by ink I-3 (5.6 × 10^−06^ Ω.cm), while at a temperature of 150 °C, the best result was obtained using ink I-7 (5.4 × 10^−05^ Ω.cm). Formulations I-6 and I-7 were best suited for the inkjet printing process, providing evidence that the presence of humectants (i.e. ethylene glycol and ethanolamine) and dispersants in the formulation is essential to the fabrication process. Resistivities of inks I-6 and I-7 printed on photo paper were 1.6 × 10^−04^ and 5.7 × 10^−05^ Ω.cm, respectively, which were dried at room temperature for 24 h. Thus, only these two formulations were selected to print the electrodes, which were submitted to the EIS experiments using a buffer solution of pH 7.4. The best results from the analysis of electrical impedance were obtained with electrodes patterned with ink I-7 (143.91 ± 2.87 Ω) and using design D-6. Taking into consideration all tests executed to evaluate the performance of the inks, such as printability, viscosity, resistivity, adhesion, and electrical impedance, the formulations most appropriate for flexible electronic and biologic applications were inks I-6 and I-7. Considering the excellent mechanical and electrical properties obtained with these formulations, our future work will focus on the application of these conductive materials in a fully printed sensor for biosensor application.

## Supplementary information

Supplementary Information.

## Data Availability

The data that support the findings of this study are available from the corresponding author on request.
