# Supplementary material for: Silver nanoparticle conductive inks: synthesis, characterization, and fabrication of inkjet-printed flexible electrodes
Source: Sci Rep. 2020 Jun 1;10:8878. doi: 10.1038/s41598-020-65698-3 (PMC7264204; doi:10.1038/s41598-020-65698-3)

**Supplementary Information**

**Silver nanoparticle conductive inks: synthesis, characterization and fabrication of inkjet-printed flexible electrodes**

Iara J. Fernandes^1,†,*^, Angélica F.~~.~~ Aroche^1,†^, Ariadna Schuck^1,†^, Paola Lamberty^1^, Celso R. Peter^1^, Willyan Hasenkamp^1^, Tatiana L. A. C. Rocha^1^

^1^ itt Chip - Institute of Technology on Semiconductors, Universidade do Vale do Rio dos Sinos, São Leopoldo, 93022-750, Brazil

^†^These authors contributed equally to this work.

^*^Corresponding author. Email: iarajf@unisinos.br

**Supplementary Figure S1. Glass substrate with vinyl tape (circular shape) and with deposited ink I-1 cured at 150, 200, and 300 °C for 30 min for resistivity measurements (first to forth image from the left), and glass substrate with vinyl tape (square shape) and with deposited ink I-1 cured at 150 °C for 30 min for adhesion test (last two images on the right).**


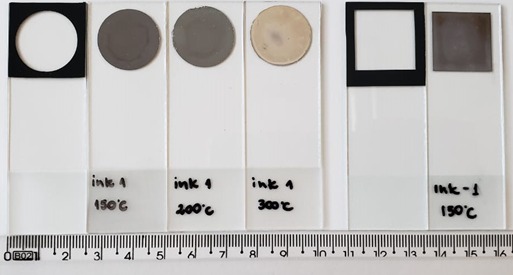


**Supplementary Figure S2. Schematic illustration of the measurement setup (2 electrodes) during the EIS assay (counter electrode (CE); reference electrode (RE); working electrode (WE)) using Phosphate-Buffered Saline (PBS) solution as analyte.**


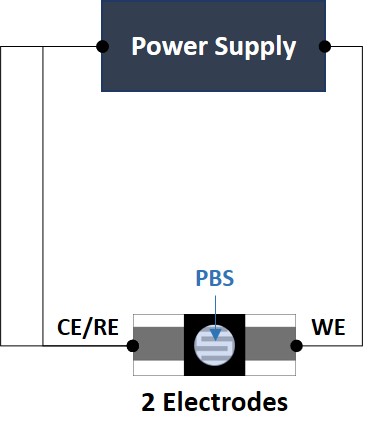

Supplement: Supplementary file 1 — Supplementary Information. [file 41598_2020_65698_MOESM1_ESM.docx]
